# Prediction of Atrial Fibrillation Using Machine Learning: A Review

**DOI:** 10.3389/fphys.2021.752317

**Published:** 2021-10-28

**Authors:** Andrew S. Tseng, Peter A. Noseworthy

**Affiliations:** Department of Cardiovascular Diseases, Mayo Clinic, Rochester, MN, United States

**Keywords:** atrial fibrillation, electrocardiogram, echocardiography, risk factor, prediction, deep learning, machine learning

## Abstract

There has been recent immense interest in the use of machine learning techniques in the prediction and screening of atrial fibrillation, a common rhythm disorder present with significant clinical implications primarily related to the risk of ischemic cerebrovascular events and heart failure. Prior to the advent of the application of artificial intelligence in clinical medicine, previous studies have enumerated multiple clinical risk factors that can predict the development of atrial fibrillation. These clinical parameters include previous diagnoses, laboratory data (e.g., cardiac and inflammatory biomarkers, etc.), imaging data (e.g., cardiac computed tomography, cardiac magnetic resonance imaging, echocardiography, etc.), and electrophysiological data. These data are readily available in the electronic health record and can be automatically queried by artificial intelligence algorithms. With the modern computational capabilities afforded by technological advancements in computing and artificial intelligence, we present the current state of machine learning methodologies in the prediction and screening of atrial fibrillation as well as the implications and future direction of this rapidly evolving field.

## Introduction

Atrial fibrillation (AF) is the most common arrhythmia worldwide with its burden expected to continue to increase with the aging population. AF is diagnosed clinically, requiring detection of the arrhythmia on formal electrocardiographic testing. Improvements in monitoring technology, including high-fidelity long-term monitors, have increased the yield for the detection of AF, thereby enhancing our knowledge of the true clinical burden of AF.

Beyond detection, there has been immense interest in prediction of AF using both clinical risk factors as well as objective testing. Numerous clinical risk scores have been proposed, incorporating readily available variables from the patient’s medical history, such as age, ethnicity, height, weight, blood pressure, smoking status, medication use, and comorbidities (Schnabel et al., 2009; Chamberlain et al., 2011; Alonso et al., 2013; Suenari et al., 2017; Li et al., 2019; Hu and Lin, 2020; Lip et al., 2020; Himmelreich et al., 2021). Abnormalities in both cardiac and inflammatory biomarkers have been shown to augment the predictive ability of clinical prediction scores (O’Neal et al., 2016). Structural cardiac abnormalities, including atrial fibrosis and atrial enlargement, as well as associated manifestations on physiologic parameters such as mitral inflow Doppler and atrial strain have been shown to be predictive of AF (De Vos et al., 2009; Caputo and Mondillo, 2012; Hwang et al., 2015). Likewise, electrocardiographic (ECG), particularly P wave morphology, has been well-studied and shown to have predictive utility. Overall, there is an abundance of clinical variables that have been shown to be predictive of AF, individually or in limited pairings.

With advancements in artificial intelligence technology and the rapid accumulation of digital clinical data, machine learning has the potential to analyze and synthesize seemingly disparate variables to predict AF in such a way that vastly surpasses conventional methods (Siontis et al., 2020). Machine learning algorithms can not only assist in processing imaging or electrocardiographic data, but it may also be able to incorporate and interpret large amounts of clinical data and discover new clinical patterns and concepts. We seek to present the latest review of conventional and machine learning methodologies in the prediction of AF.

## The Present State of Machine Learning Techniques

At the core of machine learning is the convergence of statistical analytics and computer engineering. Machine learning algorithms are able to process complex inputs, such as images, and discern subtle relationships that may not be evident with traditional statistical methods. Machine learning techniques can be categorized broadly into three categories: supervised learning, unsupervised learning, and reinforcement learning. Supervised learning requires labels during training, such as the presence or absence of incident AF. Therefore, the algorithm is provided with both the input variables as well as outcome labels. Unsupervised learning seeks to identify relationships within the data without the assistance of labels. Various methods such as clustering have been described for this method of learning. Reinforcement learning uses the concept of reward maximization, in which the machine learning algorithm assumes the role of an agent that receives either positive or negative reinforcement to guide decision making (Thrun and Littman, 2000; Koohy, 2017; Géron, 2019). For the purposes of this review, we will discuss the most used learning method, supervised learning.

Supervised learning itself utilizes different methods including regression modeling, random forests, and neural networks. In regression modeling, both with and without machine learning, preselected variables undergo regression analysis to determine their ability to predict an outcome. Machine learning improves upon these traditional modeling techniques by its ability to analyze large and complex datasets. Techniques include classification algorithms such as Support Vector Machine and K-Nearest Neighbor (Sultana et al., 2016). Random forests utilize branching decision trees, empirically deriving thresholds to determine how the data should be split (Koohy, 2017; Géron, 2019; Uddin et al., 2019).

Neural networks have fundamentally changed the machine learning landscape. Fundamentally, the network architecture is comprised of layers and processing units within each layer called nodes. Data is analyzed in one layer and then transmitted to the next layer, such that a node in a deeper layer receives inputs from one or more nodes in the prior layer. All neural networks have an input layer to process input data and an output layer while a deep neural network continues numerous “hidden” intermediary layers and nodes. Convolutional neural networks utilize the concept of “convolutions,” whereby nodes in a deeper layer only receive input from select subset of nodes from the previous layer. Therefore, these networks seek to identify local correlations and preserve local special dependences, which is particularly important for image processing. It also allows for more efficient computational processing by reducing the input data into smaller localized convolved features via methods of dimensionality reduction (Stankovic and Mandic, 2021).

There are a vast array of different machine learning techniques, which by themselves can be the subject of reviews and textbooks. For the clinician, we have summarized the different supervised machine learning techniques, including names of techniques one might encounter, as well as the general advantages and disadvantages in Table 1.

**TABLE 1:** Advantages and disadvantages of different supervised machine learning techniques.

Examples	Advantages	Disadvantages
Linear/logistic regression	Simple and easy to implement	Reduced accuracy with variables with complex relationships
k-Nearest Neighbor	Simple and can handle noisy instances or instances with missing attribute value	Computationally taxing, does not identify variables that are important for classification
Support vector machines	More robust than ordinary regression and can classify semi-structured/unstructured data	Computationally taxing, does not handle data noise well
Random forests	Performs well in large datasets and identifies variables that are important for classification	Computationally taxing, easy to overfit
Neural networks	Detects complex non-linear relationships between variables	Computationally taxing, cannot access decision making process (“blackbox”)

## From Clinical Data

Validated clinical risk scores to predict AF, such as the FHS, ARIC, CHARGE-AF, C2HEST, and HATCH score, utilize readily obtainable clinical variables, such as age, ethnicity, height, weight, blood pressure, smoking status, antihypertensive medication use, history of diabetes, heart failure myocardial infarction, etc. Based on these readily available variables from the patient history, these risk scores have shown adequate model discrimination for the prediction of incident AF (area under the receiver operator curve, AUCs, generally around 0.70) (Schnabel et al., 2009; Chamberlain et al., 2011; Alonso et al., 2013; Suenari et al., 2017; Li et al., 2019; Hu and Lin, 2020; Lip et al., 2020; Himmelreich et al., 2021). AUCs, or c-statistic, are commonly used in studies of diagnostic test performance as an overall indicator of test performance (Bradley, 1997). Other measures of test performance, though not universally reported, include accuracy (proportion of correct assessments), precision (or positive predictive value), and recall (or sensitivity). Due to inconsistencies with reporting these other measures of test performance, which limits comparison among studies, we will largely focus on AUCs. The studies for these validated clinical risk scores to predict AF are summarized in Table 2.

**TABLE 2:** Original and validation studies of clinical AF risk scores.

Clinical AF risk score	Original study	Example validation*
**FHS** (Age, sex, BMI, SBP, hypertension treatment, PR interval, clinically significant cardiac murmur, CHF)	4,764 patients from the United States AUC 0.78 (95% CI 0.76–0.80) (Schnabel et al., 2009)	49,599 patients from the United States 0.734 (0.724–0.744) (Shulman et al., 2016)

**ARIC** (Age, race, height, SBP, hypertension treatment, smoking status, precordial murmur, left ventricular hypertrophy, left atrial enlargement, DM, CAD, CHF)	14,546 patients from the United States AUC 0.765; 95% CI, 0.748–0.781 (Chamberlain et al., 2011)	None

**CHARGE-AF** (Age, ethnicity, height, weight, blood pressure, smoking, antihypertensive use, DM, CHF, MI)	18,556 patients from the United States AUC 0.765 (95% CI: 0.748–0.781) (Alonso et al., 2013)	114,475 patients from the Netherlands AUC 0.74 (95% CI: 0.73–0.74) (Himmelreich et al., 2021)

**C2HEST** (CAD/COPD, hypertension, age, CHF, hyperthyroidism)	471,446 patients from China AUC 0.75 (95% CI: 0.73–0.77) (Li et al., 2019)	1,047,330 patients from Denmark AUC 0.588 (95% CI: 0.585–0.591) (Lip et al., 2020)

**HATCH** (Hypertension, age, stroke/TIA, COPD, CHF)	670,804 patients from Taiwan AUC 0.716 (95% CI: 0.710–0.723 (Suenari et al., 2017)	692,691 patients from Taiwan AUC 0.771 (no CI provided) (Hu and Lin, 2020)

*AF, atrial fibrillation; AUC, area under the curve; BMI, body mass index; CAD, coronary artery disease; CHF, congestive heart failure; COPD, chronic obstructive pulmonary disease; DM, diabetes mellitus; MI, myocardial infraction; SBP, systolic blood pressure; TIA, transient ischemic attack.*

*Studies selected for explanatory purposes and may not be an exhaustive list.

The addition of serologic testing of common cardiac biomarkers, including natriuretic peptides and C-reactive protein, has been shown to enhance the predictive ability of such clinical risk scores (Sinner et al., 2014). Additional markers of chronic kidney disease, such as Cystatin C, and endothelial dysfunction have also been shown to be associated with AF, though no studies have been shown that the addition of these parameters enhances the predictive ability of existing clinical risk scores (O’Neal et al., 2016).

With the abundance of clinical and laboratory data available in digital format, recent investigators have started to evaluate the use of machine learning in predicting AF using the electronic health record. To facilitate this, organizations have developed a common data models for analysis, one prime example being the Observational Medical Outcomes Partnership Common Data Model in an effort to synchronize data from disparate sources for systematic analysis (FitzHenry et al., 2015). In a recent large study of nearly 2 million patients from the University of Colorado health systems by Tiwari et al. (2020) investigators applied a machine learning model to over 200 most common health record features, including demographics and comorbidity data, and derived a model with an AUC of 0.79 to detect incident AF in a 6 month timeframe, which is in line with non-machine learning clinical AF risk scores. In another study of over 2 million primary care patients from the United Kingdom by Sekelj et al. (2021) another machine learning algorithm achieved an AUC of 0.83 in the development dataset and 0.87 in the validation dataset to detect incident AF in a registry that spanned 7 years, indicating better performance compared to traditional risk scores.

When comparing the AI algorithms to the traditional risk scores, many factors may impact and limit the interpretation of the test performance. Firstly, there is significant variation in the duration of follow-up for each study, ranging from as short as 6 months to more than 10 years. This clearly significantly impacted the proportion of patients at study termination with incident AF (1% vs. 10%, respectively) (Schnabel et al., 2009; Tiwari et al., 2020). It is possible that limited follow-up such as 6 months in the Tiwari AI study may have reduced the test performance in part due to the limited duration of follow-up, where “false positives” (i.e., AI screening positive, AF negative at 6 months) would have been “true positives” if given sufficient time to manifest or vice versa with “true negatives” at the end of study turning into “false negatives” (Tiwari et al., 2020).

In a recent study by Hill et al. (2019) of nearly 3 million patients in the United Kingdom, the investigators compared a machine learning algorithm with time-varying covariates to the CHARGE-AF risk score. The use of time-varying covariates represents yet another technique in neural networks, in which the input covariates are not static but are allowed to be incorporated into the model at varying time points during the study period. This means that the temporal association between a covariate and the outcome becomes another critical factor during the development of these neural networks. In this study, the found that the time-varying model had an AUC of 0.827 while the traditional CHARGE-AF risk score applied to the same population had an AUC of 0.725. Using the time-varying methodology, they were able to determine that congestive heart failure diagnosed within the most recent 91-day quarter contributed the most to the prediction of incident AF. This study not only showed the benefits of using different machine learning techniques to extract potentially clinically relevant predictors (such as time-dependent variables), but also that the machine learning algorithms performed more robustly than traditional risk scores (Hill et al., 2019). While these algorithms have not been tested prospectively nor have they been validated in external health systems, the size and scale of these massive projects far exceed previous studies of conventional clinical risk scores for AF, and shows the increasing promise of utilizing easily accessible data from the electronic health record to predict the risk of incident AF.

## From Cardiac Imaging Data

AF is often associated with distinct structural heart abnormalities that are apparent on cardiac imaging, including echocardiography, CT and MRI. Oftentimes, these structural abnormalities result from conditions that predispose patients to AF, such as diastolic dysfunction, but AF can also itself lead to valvular regurgitation. From an echocardiography perspective, previous studies have shown that left atrial volumes, measures of diastolic dysfunction, ventricular wall thickness, strain echocardiography can be associated with the risk of new-onset AF (Xu et al., 2011; Caputo and Mondillo, 2012; Hirose et al., 2012). Newer, non-conventional measurements such as the total atrial conduction time, as a marker of atriopathy, was also shown to be associated with development of AF in a smaller cohort of 249 patients (De Vos et al., 2009). Cardiac CT to evaluate the left atrial appendage have demonstrated mixed results on prediction of AF after AF ablation (Ebersberger et al., 2020). However, left atrial thickness as a marker of atriopathy on cardiac CT has been shown to be associated with increased risk of transition for paroxysmal AF to chronic AF as well as low-voltage areas as potential sites of ablation (Nakamura et al., 2011; Nakatani et al., 2020). Given the unique ability of MRI to evaluate tissue characteristics, left atrial fibrosis by late gadolinium enhancement on cardiac MRI has been shown to be associated with new-onset AF. One study with 182 patients evaluated the predictive ability of left atrial fibrosis>6% and derived an AUC of 0.67, which was further enhanced to 0.80 after adding history of hypertension and left ventricular ejection fraction (Siebermair et al., 2019). Overall, the use of these imaging parameters to predict AF have largely been restricted to small association and procedural studies, and there has not been systematic use of imaging data to develop or refine existing risk scores for predicting AF.

Machine learning has likewise begun to make headway in image analysis. Unlike the categorical or numerical input of data from the electronic health records, images require additional sophisticated methodologies when applying machine learning, yet the fundamental theory remains similar (Fu et al., 2019). Small-scale studies have started to investigate the use of machine learning on cardiac imaging. In a small study on cardiac CT using machine learning to evaluate left atrial and pulmonary vein morphology in 203 patients undergoing AF ablation, the machine learning algorithm was able to predict AF recurrence after ablation using these CT images with an AUC of overall AUC of 0.87 (Firouznia et al., 2021). A similar study of 68 patients using cardiac CT left atrial morphology to predict AF recurrence after ablation demonstrated an AUC of 0.78 when combining imaging and clinical features (Atta-Fosu et al., 2021).

However, there have not been investigations in the use of machine learning in cardiac imaging to predict new-onset AF. Given multiple factors, including the complexity of image processing, machine learning in cardiac imaging has focused on image acquisition, processing, and basic interpretation (Chang et al., 2020). Future studies will be needed to develop the role of machine learning in prognosis and detection of non-imaging diagnoses such as AF. As such, large population-based studies may not be feasible, related to the costs of screening asymptomatic patients with imaging and significant selection bias for patients who have indications to undergo cardiac imaging tests. Nonetheless, machine learning in cardiac imaging for AF will undoubtedly play an important role in periprocedural prognosis and management, and perhaps with well-designed studies can help with the prediction of AF.

## From Electrophysiological Data

Pathophysiologic changes in AF can also manifest itself as abnormalities on electrophysiology testing, such as electrocardiography and invasive intracardiac electrograms. Previous studies have shown that ECG findings, such as P-wave duration, dispersion and amplitude as well as premature atrial contraction morphology and frequency, have been shown to be predictive of incident AF, achieving AUCs ranging from 0.69 to 0.87 (Dilaveris et al., 2000; Thong et al., 2004; Yoshizawa et al., 2014). One study evaluated premature atrial contraction characteristics and percent burden as a risk factor for AF among 652 patients who underwent Holter monitoring, with an AUC of 0.58 (Im et al., 2018).

Intracardiac electrograms are generally obtained during an electrophysiology study in patients with known or suspected arrhythmia. Therefore, there have not been studies specifically evaluating the predictive ability of electrogram features on new-onset AF. However, elements of the intracardiac electrogram have been shown to be correlated with the risk of AF recurrence after ablation. For example, in one study on 140 patients, multiple electrogram characteristics including dominant frequency, regularity index and organizational index of fibrillatory electrograms have shown predictive value for AF recurrence after AF ablation (Szilagyi et al., 2018).

The use of machine learning on the ECG to predict new-onset AF has been the subject of immense inquiry recently. Unlike the use of machine learning to process cardiac imaging, the processing of electrocardiographic signals is highly standardized using a static time series dataset and more easily interpretable compared to a series of images, including moving images like in echocardiography. In a large study from the Mayo Clinic of over 600,000 ECGs in normal sinus rhythm, a convolutional neural network was developed with a robust AUC of 0.87 for predicting new-onset AF, with further augmentation of the AUC to 0.90 for patients with multiple ECGs (Attia et al., 2019). A small study by Ebrahimzadeh et al. (2018) in 53 patients of extended ECG recordings sought to evaluate different machine learning techniques using heart rate variability analysis in extended ECG monitoring to predict initiation of AF. In this self-controlled study, all patients had an episode of paroxysmal AF, in which a 5-min ECG segment obtained 30 min prior to the onset of AF (“AF” label) was compared to a 5-min ECG segment obtained 45 min after termination of AF (“non-AF” label). Unlike the convolutional neural network used in the Mayo Clinic study in which the features of the neural network are hidden, the investigators identified multiple features from the heart rate variability signal, including linear, non-linear and time frequency features, in order to develop the machine learning model. They found that the combined machine learning approach performed better than traditional machine learning classifiers (Multilayer Perceptron, K-Nearest Neighbor, Support Vector Machine) (accuracy of 98.21% vs. 91.90–93.76%, respectively) (Ebrahimzadeh et al., 2018).

Thus, there are numerous techniques in machine learning being explored for the use of electrophysiological data to predict AF. These techniques range from different “traditional” machine learning classification algorithms to convolutional neural networks. No direct comparison between traditional models and machine learning models in ECG interpretation have been performed to date. However, machine learning methodologies allow analysis of large quantities of ECG data that may be too cumbersome and time consuming to perform manually and has thus far allowed for the development of prediction models with strong diagnostic performance.

## Future Direction

As the reader considers the various sections in this review from clinical data to electrophysiological data, we can see that machine learning, while still in its infancy, has begun to drastically improve our ability to predict AF. There are current worldwide efforts and clinical trials to prospectively test and harness the potential of AI in clinical practice for AF. In the United States, the Batch Enrollment for AI-Guided Intervention to Lower Neurologic Events in Unrecognized AF, or BEAGLE trial, seeks to assess the performance of AI on detecting AF on normal sinus ECGs in adult patients who do not have a previous diagnosis of AF and are eligible for anticoagulation based on standard stroke risk stratification (Yao et al., 2021). Similar efforts are being undertaken in France, United Kingdom, the Netherlands, Finland, and Germany, some also testing the utility of AI applied to ECGs obtained by portable devices (ClinicalTrials.gov, 2021a,b,c,d,e).

Despite these important advancements, there is still significant room for growth within this space.

(1) Integration of all modalities of data: While siloed approaches are often necessary in the beginning to refine specific methodologies as it pertains to different modalities of data, we have seen from conventional studies that the combination of data (e.g., clinical, laboratory, imaging, etc.) often leads to the highest predictive ability for any clinical risk score. This same principle should be applied to machine learning algorithms, such that the development of a machine learning algorithm that can incorporate all modalities of data, likely further enhancing the powerful predictive performance of the existing AI algorithms.
(2) Advancements in our understanding and methodologies of machine learning: At this early stage, due to the nature of many advanced types of machine learning, including convolutional neural networks, the signal features selected by the AI as important predictive features in an algorithm cannot be known (the so-called “black box”). It is possible that future techniques will allow the algorithms to be more explicit and informative about its own methodologies, both to inform clinicians on novel patterns that may advance human understanding but also to inform researchers on potential troubleshooting issues, such as the inadvertent use of non-medical or unrelated data in their predictive algorithms.
(3) Implications of machine learning algorithms on management: While the overall aim of this review is to evaluate the role of AI in predicting AF, future studies should undoubtedly evaluate the prospective use of these algorithms to determine optimal management strategies for patients. In AF, for example, there is significant implication with AF diagnosis regarding stroke prevention via the use of anticoagulation. Could there be important changes to clinical outcomes and patient management based on the results of the algorithm that can be eventually be actionable, perhaps even before a clinical diagnosis?

## Conclusion

There is no doubt that artificial intelligence will play a greater role in medicine as the technology continues to advance and our understanding of its applications continues to grow. While still in its early stages and still flawed by inherent limitations, machine learning shows great promise in improving our ability to predict AF. The future integration of clinical, imaging and electrophysiological data will certainly improve the performance of these machine learning algorithms, and ultimately improve the care of patients worldwide.

## Author Contributions

PN formulated the concept and format of the article and made critical revisions to the manuscript. AT wrote and revised the manuscript. Both authors contributed to the article and approved the submitted version.

## Conflict of Interest

PN receives research funding from National Institutes of Health [NIH, including the National Heart, Lung, and Blood Institute (NHLBI, R21AG 62580-1, R01HL 131535-4, R01HL 143070-2) the National Institute on Aging (NIA, R01AG 062436-1)], Agency for Healthcare Research and Quality (AHRQ, R01HS 25402-3), Food and Drug Administration (FDA, FD 06292), and the American Heart Association (18SFRN34230146, AHA). PN was a study investigator in an ablation trial sponsored by Medtronic. PN and Mayo Clinic were involved in potential equity/royalty relationship with AliveCor. PN has served on an expert advisory panel for Optum. PN and Mayo Clinic have filed patents related to the application of AI to the ECG for diagnosis and risk stratification. The remaining author declares that the research was conducted in the absence of any commercial or financial relationships that could be construed as a potential conflict of interest.

## Publisher’s Note

All claims expressed in this article are solely those of the authors and do not necessarily represent those of their affiliated organizations, or those of the publisher, the editors and the reviewers. Any product that may be evaluated in this article, or claim that may be made by its manufacturer, is not guaranteed or endorsed by the publisher.
